# Health-related quality of life anticipated with different management strategies for paediatric febrile neutropaenia

**DOI:** 10.1038/bjc.2011.213

**Published:** 2011-06-21

**Authors:** S Cheng, O Teuffel, M C Ethier, C Diorio, J Martino, C Mayo, D Regier, R Wing, S M H Alibhai, L Sung

**Affiliations:** 1 Department of Paediatrics, University of Toronto, 27 King's College Circle, Toronto, Ontario, Canada M5S 1A1; 2 Program in Child Health Evaluative Sciences, The Hospital for Sick Children, 555 University Avenue, Toronto, Ontario, Canada M6G 1X8; 3 Division of Haematology/Oncology, The Hospital for Sick Children, 555 University Avenue, Toronto, Ontario, Canada M6G 1X8; 4 Department of Internal Medicine, University of Toronto, 27 King's College Circle, Toronto, Ontario, Canada M5S 1A1; 5 Department of Medicine, University Health Network, 190 Elizabeth Street, Toronto, Ontario, Canada M5G 2C4

**Keywords:** health-related quality of life, child, febrile neutropaenia, willingness to pay, visual analogue scale, time trade-off technique

## Abstract

**Background:**

To describe (1) anticipated health-related quality of life during different strategies for febrile neutropaenia (FN) management and (2) attributes of those preferring inpatient management.

**Methods:**

Respondents were parents of children 0–18 years and children 12–18 years receiving cancer treatment. Anticipated health-related quality of life was elicited for four different FN management strategies: entire inpatient, early discharge, outpatient oral and outpatient intravenous (i.v.) therapy. Tools used to measure health-related quality of life were visual analogue scale (VAS), willingness to pay and time trade off.

**Results:**

A total of 155 parents and 43 children participated. For parents, median VAS scores were highest for early discharge (5.9, interquartile range 4.4–7.2) and outpatient i.v. (5.9, interquartile range 4.4–7.3). For children, median scores were highest for early discharge (6.1, interquartile range 4.6–7.2). In contrast, the most commonly preferred strategy for parents and children was inpatient in 55.0% and 37.2%, respectively. Higher current child health-related quality of life was associated with a stronger preference for outpatient management.

**Conclusion:**

Early discharge and outpatient i.v. management are associated with higher anticipated health-related quality of life, although the most commonly preferred strategy was inpatient care. This data may help with determining more cost-effective strategies for paediatric FN.

Febrile neutropaenia (FN) continues to be a frequent complication of chemotherapy in children with cancer despite advances in supportive care. Due to the potential for sepsis, the standard therapy in children has been inpatient management with broad-spectrum intravenous (i.v.) antibiotics (Bodey *et al*, 1966; Hughes *et al*, 1990; Caggiano *et al*, 2005). It is now well recognised, however, that only a small proportion of patients develop serious medical complications (Klastersky *et al*, 2000). There is increasing evidence that adults and children with low-risk FN can be treated with alternate management strategies such as early discharge or outpatient therapy (Dommett *et al*, 2009). These alternate strategies are attractive for a number of reasons, including the reduced risk of nosocomial infection and better resource management, as the major financial burden of conventional FN management is the cost of inpatient care (Leese, 1993; Rubenstein *et al*, 1993; Dranitsaris *et al*, 1995). In addition, it has been hypothesised that outpatient management may increase child health-related quality of life (HRQL) (Mustafa *et al*, 1996; Mullen *et al*, 1999).

While anticipated differences in HRQL are one motivation for alternate FN strategies, little research has documented such differences, particularly in the paediatric setting where the perspectives of both the parent and child may be important. Consequently, we were interested in exploring parent and patient anticipated HRQL in, and preferences for, different treatment options for low-risk FN. One potential application of this data would be the incorporation into a cost-utility analysis, and thus, we were interested in obtaining utilities. Utility can be defined as the strength of an individual's preference for a health state when measured under conditions of uncertainty, and was established through von Neumann and Morgenstern's modern utility theory (VonNeumann and Morgenstern, 1953; Torrance, 1987). While the standard approach to utility elicitation is the standard gamble, we chose to use alternate approaches to estimate utility, namely visual analogue scale (VAS), willingness to pay (WTP) and time trade off (TTO), as the standard gamble is more complex and difficult to administer (Morimoto and Fukui, 2002; Ross *et al*, 2003). While VAS is not a utility, we included this measure as VAS may be related to utility in a non-linear fashion (Torrance *et al*, 2001).

In addition to estimating HRQL, we were also interested in determining the most commonly preferred strategy from the perspective of parents and children. This information would shed insight into the relationship between anticipated HRQL and treatment preferences. Also, we were interested in identifying which families would prefer inpatient management, as this information could aid in outpatient programme development.

Therefore, the primary objective was to describe anticipated HRQL during different strategies of FN management using VAS, WTP and TTO from the perspective of parents of children with cancer and older children themselves. The secondary objective was to describe attributes of those who preferred inpatient management of FN.

## Materials and methods

### Participants

We included two groups of respondents: (1) parents of children 0–18 years of age and (2) children 12–18 years of age. To be eligible, (a) the child had to be receiving active treatment for cancer and had to have presented to The Hospital for Sick Children in Toronto, Canada, for any reason and (b) respondents had to be able to read English and be able to provide informed consent. Those admitted for haematopoietic stem cell transplant were excluded.

### Study design

The study was approved by the Research Ethics Boards at The Hospital for Sick Children and all respondents provided informed consent. We recruited eligible participants in a consecutive fashion from the outpatient clinics and inpatient units. Trained research assistants conducted face-to-face interviews with the respondents using standardised scripts and visual aids, which were both extensively pilot tested prior to implementation.

**Figure 1:**
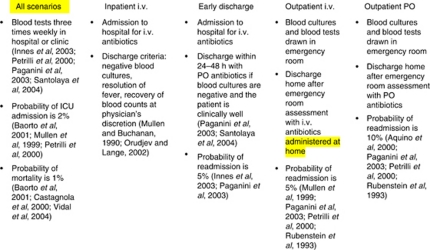
Attributes associated with the four FN scenarios.

The primary objective was to describe HRQL during four different FN management strategies from parent proxy respondents who anticipated HRQL on behalf of their child and from children who responded on their own behalf. We considered the four different options for FN management that we believed most likely to be adopted in clinical practice in paediatric oncology. These options were (1) entire inpatient management with i.v. antibiotic administration; (2) early discharge (discharge within 24–48 h) with initial inpatient i.v. antibiotic administration followed by outpatient oral (PO) antibiotic administration; (3) entire outpatient management with i.v. antibiotics; and (4) entire outpatient management with PO antibiotic administration. Although inpatient management with PO antibiotics and early discharge with i.v. antibiotics are also possible strategies, we believe they are less likely to be adopted and they were, therefore, not included. The attributes and the probabilities of outcomes such as readmission, intensive care unit admission and mortality were derived from a review of the literature, and these attributes, probabilities and citations are shown in Figure 1.

Respondents were asked to imagine that their child or they themselves had low-risk FN that could be treated in one of the four ways previously described. It was emphasised that these scenarios were hypothetical and that some management options might not be appropriate for their child/themselves. First, respondents were asked to rank the four options in terms of preference. Then, respondents were asked to estimate HRQL with the four different FN management strategies using the VAS, WTP and TTO as described below. The order of administration was constant for all respondents.

### Outcome measures

#### Visual analogue scale

Participants were asked to estimate their child's/their own anticipated HRQL for each strategy by drawing a vertical line across a horizontal 10 cm VAS anchored at the left end by the worst possible HRQL (score of 0) and at the right end by perfect HRQL (score of 1).

#### Willingness to pay

Another measure of a respondent's preference for a management strategy is how much he/she would be willing to pay to receive that strategy. We obtained relative WTP such that inpatient i.v. antibiotics was considered the standard free option and asked respondents how much money, in Canadian dollars, they would be willing to pay out-of-pocket to switch from inpatient i.v. antibiotics to one of the three alternative presented strategies. We used a visual sliding bar tool with anchoring amounts of $0 and $1000 as a presentation aid to help respondents conceptualise the exercise. Starting with the early discharge option, parents/children were asked if they would choose this option over inpatient i.v. therapy if there were no costs. If the respondent replied no, the WTP was recorded as $0. If the respondent replied yes, he/she was asked if he/she would choose early discharge if this option costs $1000. If they responded yes, we asked them the maximum amount they would pay for early discharge. If they said no, we used a ping-pong, then a titration approach, to determine the maximum amount they would be willing to pay for the alternative management strategy, early discharge. This process was repeated with the remaining two treatment options.

#### Time trade off

Time trade off asks respondents to compare different combinations of quantity and quality of life. Using a visual aid board, the point at which respondents are indifferent to a choice between a scenario in which they would live a specified time period in a hypothesised or actual health state or a second scenario in which they would live in perfect health but for a length of life less than the first scenario can be determined. The amount of time the respondent is willing to trade off for perfect health reflects their utility or estimate of HRQL for the health state under consideration.

We initially attempted to obtain absolute values of TTO for the four different FN options. However, given the short-term nature of FN, pilot testing demonstrated that respondents were unable to conceptualise absolute TTO for the four different FN strategies. Consequently, TTO values were elicited in a similar manner to WTP. Respondents were instructed that they were going to go through a similar series of steps as WTP, but instead of asking respondents how much money they would be willing to pay to switch options, we would ask them how much time they would be willing to give up from the remaining length of life to receive the alternative treatment strategy. A similar sliding bar scale was used with anchors of 0 years at one end and 50 years at the other end.

Participants were first asked if they would be willing to give up 1 day of their child's/their own life to switch from the inpatient i.v. treatment to early discharge. If participants said no, their answer was recorded as 0. If they said yes, they were then asked if they would give up the remaining years of their child's/their own life to switch treatment options. It was understood that all respondents would say no unless they believed that receiving inpatient i.v. treatment was worse than death. The time given up to receive early discharge was then sequentially altered using a ping-pong, then a titration approach until the respondent was indifferent to the choice. This was marked as the maximum time the respondent would be willing to give up in order to receive early discharge for one episode of FN. Typically, TTO is presented as a utility, which is a number that ranges from 0 to 1. However, given that respondents could not conceptualise absolute TTO and the time frame being given up was in the order of days to weeks rather than years, we have presented this value as the number of weeks given up rather than as a utility. This process then was repeated for the other two treatment strategies.

### Predictors of preferences

We also wanted to determine predictors of preferences for inpatient management. The following were examined as potential predictors: parent variables consisting of demographics, socioeconomic variables, time to travel to the hospital and previous experience with FN; and child/cancer variables consisting of demographics, time since diagnosis and underlying cancer type. In addition, we included the child's current HRQL using two instruments, namely VAS and the health utilities index (HUI). Visual analogue scale was administered similar to that described above. The HUI is a family of multi-attribute health status classification systems that currently consist of two complementary systems, HUI2 and HUI3 (Furlong *et al*, 2001). Health utilities index 2 is composed of seven attributes as follows: sensation, mobility, emotion, cognition, self-care, pain and fertility. Health utilities index 3 is composed of the eight attributes as follows: vision, hearing, speech, ambulation, dexterity, emotion, cognition and pain. Health states defined by a comprehensive set of HUI levels can be used to determine single attribute and overall utility scores. We used an interviewer-administered questionnaire comprised of 41 items that assessed health status over the most recent 1 week time frame. Proxy-respondent completed HUI is only available for children 5 years and older; and thus, only 84 parent respondents provided HUI scores. Proxy-report HUI is reliable and valid in paediatric cancer (Sung *et al*, 2003).

### Statistics

The primary objective was descriptive. In order to compare the four different management strategies for VAS, WTP and TTO within each respondent type, we conducted repeated measures linear regression. We did not compare parent and child responses as some of these respondents were from the same family and it would not have been appropriate to consider them as independent groups. Furthermore, the matched subset was too small for such a comparison. For the secondary objective, we examined the proportion of parents and children who ranked inpatient management first and subsequently conducted univariate logistic regression to determine predictors of preference for inpatient management for parent respondents only given the limited sample size in the child self-respondent group. All statistical analyses were performed using the SAS statistical program (SAS-PC, version 9.1; SAS Institute Inc., Cary, NC, USA). All tests of significance were two sided, and statistical significance was defined as *P*<0.05.

## Results

From 4 June 2009 to 29 December 2009, a total of 210 potential parents were identified for participation in the study. Of these, 28 did not meet inclusion criteria and 27 declined to participate. Consequently, 155 parents participated. There were 53 potential child respondents who were approached. Of these, 10 refused, leaving 43 child respondents who participated in the study. Fifteen participants from each group were from the same family. Demographics, disease characteristics and child's current HRQL for the parent and children respondents are presented in Table 1.

Table 2 illustrates parental perspectives on their child's anticipated HRQL as measured by the VAS for the four different FN management strategies as well as WTP and TTO relative to inpatient management. From a parental perspective, outpatient PO management had the lowest VAS score, while median VAS scores for early discharge and outpatient i.v. were highest. Similarly, parents would be willing to pay some amount of money to have early discharge or outpatient i.v.; although typically, they would only pay $50 and $20, respectively, for one episode of FN. Most parents would not be willing to give up any time for an alternate management strategy for FN.

Table 3 illustrates these same variables from the child's perspective. Median VAS was not significantly different between the strategies, although the raw scores were highest for early discharge. Children were willing to pay money for any alternate management strategy compared with inpatient care, and in contrast to parents, most would give up 7–11 weeks in order to be treated with a non-inpatient management strategy.

In terms of highest-ranked strategies, most parents preferred inpatient management (80 out of 154, 52.0%) followed by early discharge (38 out of 154, 24.7%), outpatient i.v. (26 out of 153, 17.0%) and last outpatient PO (10 out of 153, 6.5%) (some respondents did not rank all strategies). For children, the highest-ranked strategy was also inpatient i.v. (16 out of 43, 37.2%), followed by home PO (11 out of 43, 25.6%), early discharge (10 out of 43, 23.3%) and last home i.v. (7 out of 43, 16.3%). Predictors of parental preference for inpatient management are illustrated in Appendix A. Two types of variables were associated with preference for inpatient management. First, parents with a higher household income were significantly less likely to prefer inpatient management. Similarly, there was a tendency for parents with higher education to have lower preference for inpatient management. Second, higher current HRQL, as assessed by parent proxy, was significantly associated with lower preference for inpatient therapy.

## Discussion

We found that when anticipated HRQL was assessed using VAS, WTP and TTO, in general, early discharge and outpatient i.v. were associated with higher anticipated HRQL from the perspective of both parents and children. In terms of TTO, few parents would be willing to trade time for an alternate strategy, which may suggest that parental TTO may have limited applicability in a transient health state such as FN. Conversely, most children would be willing to give up about 2–3 months to receive non-inpatient treatment. In contrast to anticipated HRQL, we found that the most common preferred strategy from parents and children was inpatient i.v.

It is important to note that we assessed preferences in the following two ways: by asking respondents to describe anticipated HRQL with each strategy, and also asking them to rank their preferred strategy. These two approaches appear to have resulted in different optimum strategies. Perhaps this finding is not surprising since the preferred strategy may not be the one with the highest anticipated HRQL, since other considerations such as feeling safe, convenience and costs could also be contributing to the choice of the most preferred strategy. Quezada *et al* (2007) evaluated common barriers to outpatient management of FN and found barriers to also include serious medical comorbidities, language, distance of residence from medical centre and lack of interest.

One consistent finding was that parents viewed outpatient PO as the strategy associated with the worst HRQL, and this was also their least commonly preferred option. This finding suggests that provision of oral antibiotic therapy may be an obstacle to outpatient management from the parent perspective. This may be related to uncertainty as to whether their child can or will tolerate oral medications when the child is unwell, potentially resulting in inadequate treatment and readmission.

The only two significant predictors of preferences for non-inpatient management were higher household income and higher current child HRQL. There are many pathways through which higher socioeconomic status could be associated with greater preference for early discharge or outpatient management, including greater comfort with the ability to provide care at home or greater access to resources to facilitate non-inpatient care. It is interesting that higher current HRQL as assessed by parent proxy was also significantly associated with a stronger preference for outpatient therapy. It is possible that parents of children who are unwell factor the additional complexity of outpatient care in their decision making. It is also possible that parents of children with poor HRQL did not feel that their child would benefit from outpatient therapy in terms of participation in their normal activities of daily living.

There is one other study that examined preferences for different management strategies in paediatric FN. That study compared preferences for only two options, namely inpatient i.v. *vs* outpatient PO antibiotic therapies, from parents of children with cancer (Sung *et al*, 2004). Similar to our study, they found a large number of parents (47%) preferred inpatient management. However, in contrast to our study, that study did not find an association between household income and current child HRQL with preference for inpatient therapy. Possible explanations for this discrepancy include a much larger sample size in our study and different methodology in that our study examined a dichotomous choice as the outcome, whereas the previous study examined strength of preference as the outcome.

Our results must be interpreted in light of several limitations. First, our centre used only inpatient therapy and alternate strategies were not used when this study was conducted. Experience with alternate strategies could change parent and child perspectives. Second, we only included English-speaking participants and perspectives of non-English-speaking families could be very different. Third, the majority of parent respondents (76.8%) were mothers who may have had different preferences than fathers. Fourth, we included all patient types in our studies and did not restrict our sample to diagnoses compatible with low-risk FN. However, child diagnosis type did not appear to impact on the preferred strategy. Finally, most participants were recently diagnosed and 50% of the child participants did not have a history of FN. These factors could both affect perception of HRQL and preferences for different therapeutic options.

Our study has demonstrated that from the parent perspective, anticipated HRQL is highest with early discharge and outpatient i.v., and is not concordant with the most preferred strategy, which was inpatient i.v. This data suggest that perspectives on alternate management strategies in paediatric FN are complex and respondents likely consider multiple factors when deciding on a preferred strategy. These HRQL estimates can be used for subsequent cost-utility analyses in paediatric FN. Future work should focus on programme development that can facilitate non-inpatient management. Qualitative approaches may be able to shed further insight into what factors respondents consider when choosing a preferred strategy for management of FN.

## Figures and Tables

**Table 1:** Demographics of study cohorts

	**Parent respondent *N*=155**	**Child respondent *N*=43**
*Respondent variables*		
Median age in years (IQR)^a^	38.5 (34.5, 43.5)	15.4 (13.4, 16.9)
No. male (%)	36 (23.2)	23 (53.5)
No. married (%)^b^	131/153 (85.6)	
No. minimum education (%)		
Professional/graduate	22 (14.2)	
College/university	94 (60.6)	
High school	36 (23.2)	
Primary/middle school	1 (0.6)	
Other	2 (1.3)	
No. full time employment (%)	79 (51.0)	
No. private health insurance (%)	125 (80.6)	
No. annual income ⩾$60 000 (%)^b^	67/154 (46.2)	
Median no. minutes travel to hospital (IQR)	60 (45.0, 80.0)	
		
*Febrile neutropaenia experience and cancer variables*		
No. history of FN (%)	99 (63.9)	22 (51.2)
Median years since diagnosis (IQR)	0.5 (0.1, 1.6)	0.2 (0.1, 0.6)
No. cancer type (%)		
Leukaemia	79 (51.0)	9 (20.9)
Lymphoma	18 (11.6)	17 (39.5)
Solid tumour	45 (29.0)	12 (27.9)
Brain tumour	12 (7.7)	5 (11.6)
Other^c^	1 (0.6)	0 (0.0)
		
*Current HRQL*		
Median VAS score (IQR)	7.1 (4.3, 8.2)	7.2 (5.7, 8.5)
Median HUI2 score (IQR)^d^	0.8 (0.6, 0.9)	0.8 (0.7, 0.9)
Median HUI3 score (IQR)^d^	0.9 (0.6, 1.0)	0.8 (0.6, 0.9)

Abbreviations: FN=febrile neutropaenia; HRQL=health-related quality of life; HUI=health utilities index; IQR=interquartile range; VAS=visual analogue scale.

a Missing for 10 parents.

b Not all respondents answered each question.

c Other – haemophagocytic lymphohistiocytosis.

d For parent respondents: *N*=82 and 84 for HUI2 and HUI3 as HUI is not available by proxy report for children<5 years and respondents who answer ‘didn’t know’ or ‘refuse’ are considered missing; For child respondents: *N*=36 and 38 for HUI2 and HUI3.

**Table 2:** Anticipated parental respondent visual analogue scale, willingness to pay and time trade off for each of the hypothetical four different febrile neutropaenia management strategies (*N*=155)

	**Value**	***β*±s.e.**	***P-*value**
*Median VAS score (IQR)*			<0.0001^a^
Inpatient	5.6 (2.8, 8.2)	REF	REF
Early discharge	5.9 (4.4, 7.2)	0.11±0.21	0.591
Outpatient i.v.	5.9 (4.4, 7.3)	0.06±0.26	0.804
Outpatient PO	4.7 (2.3, 7.2)	−0.81±0.37	0.030
			
*Median WTP score (IQR)* b			0.0001^a^
Inpatient	0	REF	REF
Early discharge	50.0 (0.0, 200.0)	314.1±121.7	0.010
Outpatient i.v.	20.0 (0.0, 200.0)	224.2±50.6	<0.0001
Outpatient PO	0.0 (0.0, 200.0)	268.0±89.3	0.003
			
*Median TTO presented as weeks given up (IQR)*			0.464^a^
Inpatient	0	REF	REF
Early discharge	0.0 (0.0, 0.0)	0.20±0.26	0.438
Outpatient i.v.	0.0 (0.0, 0.0)	−0.07±0.38	0.845
Outpatient PO	0.0 (0.0, 0.0)	−0.30±0.53	0.569

Abbreviations: IQR=interquartile range; i.v.=intravenous; PO=outpatient oral; REF=reference; TTO=time trade off; VAS=visual analogue scale; WTP=willingness to pay.

a These *P-*values represent differences across the four groups using repeated measures analysis.

b In Canadian dollars.

**Table 3:** Anticipated child self-report visual analogue scale, willingness to pay and time trade off for each of the hypothetical four different febrile neutropaenia management strategies (*N*=43)

	**Value**	***β*±s.e.**	***P-*value**
*Median VAS score (IQR)*			0.488^a^
Inpatient	5.7 (4.1, 7.9)	REF	REF
Early discharge	6.1 (4.6, 7.2)	0.20±0.26	0.438
Outpatient i.v.	5.6 (4.6, 7.0)	−0.07±0.38	0.845
Outpatient PO	5.3 (4.3, 7.3)	−0.30±0.53	0.569
			
*Median WTP score (IQR)* b			0.004^a^
Inpatient	0	REF	REF
Early discharge	50.0 (0.0, 150.0)	241.3±98.3	0.016
Outpatient i.v.	62.5 (0.0, 250.0)	218.3±68.4	0.0008
Outpatient PO	45.0 (0.0, 185.0)	197.8±70.8	0.006
			
*Median TTO presented as weeks given up (IQR)*			0.049^a^
Inpatient	0	REF	REF
Early discharge	7.3 (0.0, 156.0)	20.9±11.4	0.069
Outpatient i.v.	10.9 (0.0, 156.0)	11.3±6.3	0.076
Outpatient PO	7.3 (0.0, 104.0)	7.4±2.8	0.010

Abbreviations: IQR=interquartile range; i.v.=intravenous; PO=outpatient oral; REF=reference; TTO=time trade-off technique; VAS=visual analogue scale; WTP=willingness-to-pay.

a These *P-*values represent differences across the four groups using repeated measures analysis.

b In Canadian dollars.

**Table A1:** Predictors of parent preference for inpatient intravenous management of paediatric febrile neutropaenia

	**OR**	**95% CI**	***P***
*Parent/family variables*			
Age in years	0.98	0.94, 1.02	0.342
Male	0.58	0.27, 1.24	0.161
Married	0.91	0.37, 2.26	0.846
Minimum education college or university	0.48	0.22, 1.02	0.057
Full time employment	0.69	0.37, 1.31	0.257
Private health insurance	0.71	0.30, 1.73	0.456
Annual income ⩾$60 000^a^	0.37	0.17, 0.81	0.013
Minutes travel to hospital	1.01	1.00, 1.02	0.135
			
*Febrile neutropaenia experience and child and cancer variables*			
History of FN	0.72	0.37, 1.39	0.330
Child age in years	0.96	0.90, 1.02	0.367
Child male	0.73	0.38, 1.40	0.284
Years since diagnosis	0.94	0.77, 1.15	0.546
Leukaemia *vs* other diagnoses	1.68	0.89, 3.18	0.110
			
*Current HRQL*			
VAS	0.80	0.70, 0.92	0.001
HUI2	0.12	0.02, 0.87	0.036
HUI3	0.17	0.04, 0.85	0.031

Abbreviations: CI=confidence interval; FN=febrile neutropaenia; HRQL=health-related quality of life; HUI=health utilities index; OR=odds ratio; VAS=visual analogue scale.

a In Canadian dollars.

## Appendix A

Table A1
